# Distinct Neural Activities in Hippocampal Subregions Revealed Using a High-Performance Wireless Microsystem with PtNPs/PEDOT:PSS-Enhanced Microelectrode Arrays

**DOI:** 10.3390/bios15040262

**Published:** 2025-04-18

**Authors:** Peiyao Jiao, Qianli Jia, Shuqi Li, Jin Shan, Wei Xu, Yu Wang, Yu Liu, Mingchuan Wang, Yilin Song, Yulian Zhang, Yanbing Yu, Mixia Wang, Xinxia Cai

**Affiliations:** 1 State Key Laboratory of Transducer Technology, Aerospace Information Research Institute, Chinese Academy of Sciences, Beijing 100190, China; jiaopeiyao21@mails.ucas.ac.cn (P.J.); jiaqianli_talk@126.com (Q.J.); lishuqi24@mails.ucas.ac.cn (S.L.); danjin16@mails.ucas.ac.cn (J.S.); xuwei201@mails.ucas.ac.cn (W.X.); wangyu21@mails.ucas.ac.cn (Y.W.); liuyu222@mails.ucas.ac.cn (Y.L.); wangmingchuan18@mails.ucas.ac.cn (M.W.); songyl@aircas.ac.cn (Y.S.); 2 School of Electronic, Electrical and Communication Engineering, University of Chinese Academy of Sciences, Beijing 100049, China; 3 Department of Neurosurgery, China-Japan Friendship Hospital, Beijing 100029, China; zhangyulian@pku.edu.cn

**Keywords:** wireless microsystem, FPGA, neural signal processing, hippocampus

## Abstract

Wireless microsystems for neural signal recording have emerged as a solution to overcome the limitations of tethered systems, which restrict the mobility of subjects and introduce noise interference. However, existing microsystems often face data throughput, signal processing, and long-distance wireless transmission challenges. This study presents a high-performance wireless microsystem capable of 32-channel, 30 kHz real-time recording, featuring Field Programmable Gate Array (FPGA)-based signal processing to reduce transmission load. The microsystem is integrated with platinum nanoparticles/poly (3,4-ethylenedioxythiophene) polystyrene sulfonate-enhanced microelectrode arrays for improved signal quality. A custom NeuroWireless platform was developed for seamless data reception and storage. Experimental validation in rats demonstrated the microsystem’s ability to detect spikes and local field potentials from the hippocampal CA1 and CA2 subregions. Comparative analysis of the neural signals revealed distinct activity patterns between these subregions. The wireless microsystem achieves high accuracy and throughput over distances up to 30 m, demonstrating its resilience and potential for neuroscience research. This work provides a compact, adaptable solution for multi-channel neural signal detection and offers a foundation for future applications in brain–computer interfaces.

## 1. Introduction

The ability to monitor spikes and local field potentials (LFPs) in real-time is essential for advancing research in brain–computer interfaces (BCIs) [1,2,3]. These neural signals provide researchers with critical insights into the functional states of experimental subjects’ brains, supporting analyses at both the population and single-neuron levels [4]. Over the past few decades, tethered neural recording systems have matured significantly [5], exemplified by commercial platforms such as Cerebus (Blackrock Neurotech, Salt Lake City, UT, USA) and OmniPlex (Plexon, Dallas, TX, USA). However, the reliance on physical cables imposes several critical limitations. Tethered systems restrict the mobility of experimental subjects, making them unsuitable for studies requiring naturalistic behaviors or large-scale movements [6,7,8,9]. Moreover, the presence of physical connections increases the risk of infection in chronic implant studies and introduces substantial power-line noise, which complicates the analysis of signals in the 50/60 Hz range and its harmonics [10]. In response to these challenges, wireless microsystems have emerged as a transformative solution due to their unparalleled spatial portability, and become one of the most critical trends for the future [11].

Wireless microsystems can be broadly categorized into two main approaches: application specific integrated circuit (ASIC)-based and commercial off-the-shelf (COTS)-based designs. ASIC-based solutions enable large-scale recording and on-chip signal processing [12,13,14]. A notable example is Neuralink’s custom ASIC system, which supports real-time recording across 3072 channels [12]. However, the high cost of chip design and fabrication, combined with limited flexibility to adapt to diverse experimental setups, has restricted the widespread adoption of these systems [7]. In contrast, COTS-based microsystems have emerged as the mainstream approach due to their lower development costs and greater adaptability. These systems often utilize Intan’s RHD series chips as front-end components for amplification, filtering, and analog-to-digital conversion [15,16,17]. They have been successfully validated in a variety of experimental models, ranging from rodents [17,18,19] to non-human primates (NHPs) [20,21,22,23,24,25] and even humans [6,26]. Shin et al. [16] developed an interference-free wireless microsystem capable of multi-subject data transmission in social interaction experiments, highlighting the flexibility of COTS-based designs. Additionally, many studies have focused on electrocorticography recording [15,27,28,29], which, while limited in spatial resolution compared to LFPs and spikes, provides valuable insights and serves as a reference for other electrophysiological signals. Integrating electrical [6,23,30] or optical [17,31] stimulation to create closed-loop systems represents another critical direction in this field.

Despite these advancements, both ASIC-based and COTS-based approaches face significant challenges. Many existing microsystems struggle to fully realize their potential at higher channel counts and sampling rates (e.g., 32 channels at 30 kHz) [15,16,17,20]. Most COTS-based microsystems lack on-device signal processing [15,16,32], which amplifies the burden of data transmission and increases power consumption. Additionally, while Wi-Fi has become the preferred method for wireless data transmission due to its greater range and convenience compared to infrared [20] and Bluetooth [19], its performance in terms of accuracy and throughput over varying distances remains underexplored. Furthermore, advanced microelectrode materials such as platinum nanoparticles (PtNPs)/poly (3,4-ethylenedioxythiophene) polystyrene sulfonate (PEDOT:PSS)-enhanced microelectrode arrays (MEAs) are underutilized, limiting opportunities to improve signal quality in wireless microsystems.

Hippocampal CA1 and CA2 subregions were selected as the target areas for two key reasons. First, CA2 is a relatively understudied subregion [33,34], and, to the best of our knowledge, few studies directly compared neural activity between CA1 and CA2. Second, the advantages of a wireless microsystem are most apparent in experiments that require subjects to move freely across a large spatial range. Given the hippocampus’s central role in spatial memory, focusing on these subregions aligns with the objectives of our system and paves the way for future experiments designed to explore spatially demanding behavioral paradigms.

**Figure 1:**
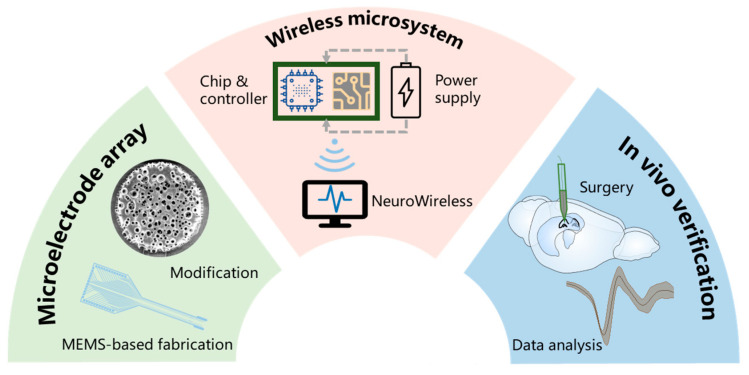
Overview of this work. The study integrates three key components. 1. Microelectrode array: fabricated using micro-electro-mechanical system technology and enhanced through surface modification. 2. Wireless microsystem: combines real-time signal processing, wireless data transmission featuring NeuroWireless, and efficient power management. 3. In vivo verification: neural signals were detected and analyzed following surgical implantation in hippocampal subregions.

As shown in Figure 1, we developed a high-performance wireless microsystem capable of real-time recording across 32 channels at a sampling rate of 30 kHz. The microsystem incorporates Field-Programmable Gate Array (FPGA)-based signal processing to reduce data transmission loads and ensure real-time, high-fidelity data transfer. Additionally, PtNPs/PEDOT:PSS-enhanced MEAs were integrated to improve signal quality, and the custom-developed NeuroWireless platform provides seamless data reception, analysis, and storage. To validate the microsystem’s capabilities, we conducted in vivo experiments in rats, successfully recording spikes and LFPs from the hippocampal CA1 and CA2 subregions. Comparative analysis of the neural signals revealed distinct activity patterns between these subregions, demonstrating the resilience and applicability of the wireless microsystem for neuroscience research.

## 2. Materials and Methods

### 2.1. Design, Fabrication, and Modification of MEA

**Figure 2:**
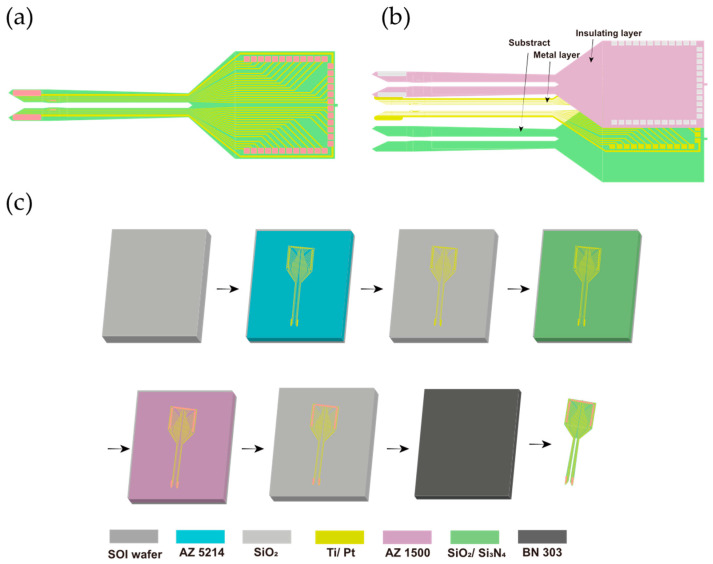
Design and fabrication of microelectrode array (MEA). (**a**) Schematic illustration of the MEA design. (**b**) Layered structure of the MEA. (**c**) The fabrication process of the MEA based on micro-electro-mechanical system (MEMS): SiO_2_ growth by thermal oxidation, metal trace patterning via photolithography and lift-off, deposition of the insulation layer, site and pad exposure by etching, and release of the MEAs through silicon etching.

The MEA probe included 36 microelectrode sites, each with a diameter of 20 μm, distributed across the surface. In total, 32 detection sites were arranged along the shape of CA1 and CA2 to maximize detection area. Among these sites, there were four sites as reference electrodes. The distance between two probes was 210 μm wide and the length of the MEA was 6.5 mm long (Figure 2a). The MEA consisted of a substrate, a metal layer, and an insulating layer (Figure 2b). Herein, the metal layer was made of Ti/Pt (30/250 μm thick), and the insulating layer consisted of SiO_2_ (700 nm thick).

All fabrication operations were performed using the micro-electro-mechanical system (MEMS) method on a silicon-on-insulator (SOI) substrate. According to our previous work [35], and illustrated in Figure 2c, the fabrication process for the MEA involved a series of steps. A thermal oxidation process was first used to grow a SiO_2_ insulating layer on an SOI wafer. Metal traces were then patterned using photolithography with AZ5214, followed by sputtering of Ti/Pt and a standard lift-off process. A SiO_2_/Si_3_N_4_ insulation layer was deposited via plasma-enhanced chemical vapor deposition, and selective etching exposed the electrode sites and bonding pads. Finally, the MEA structures were released by deep silicon etching, followed by wet etching, during which AZ1500 and BN303 were used, completing the fabrication process. After the fabrication of MEAs, detection sites were electrodeposited with PtNPs and PEDOT:PSS to enhance biocompatibility and signal-to-noise ratio [36,37].

### 2.2. Microsystem Architecture

**Figure 3:**
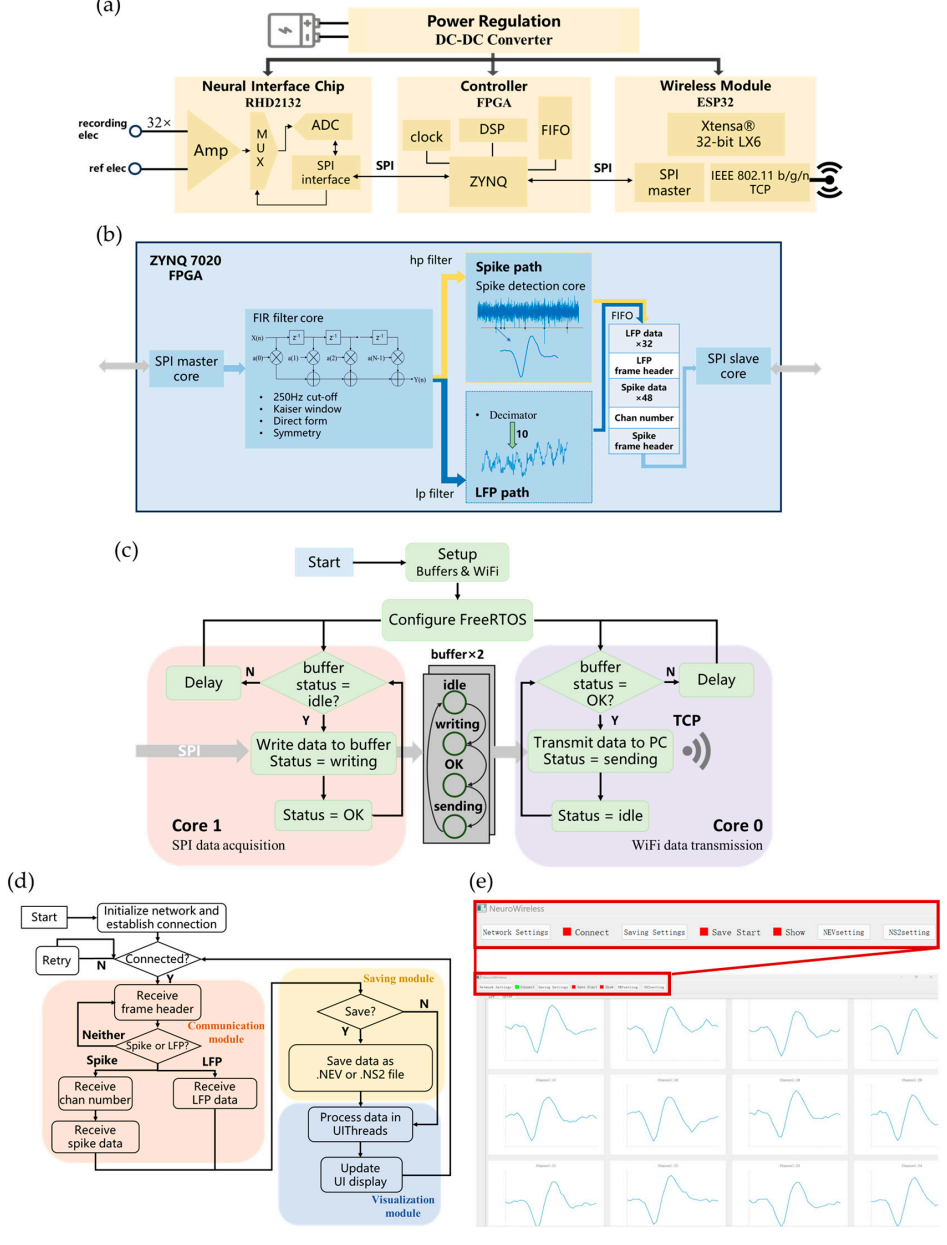
Design of the wireless microsystem. (**a**) Hardware architecture of the wireless microsystem, showing the integration of key components. (**b**) Signal flow diagram for the FPGA. (**c**) Schematic of the ESP32’s operational mechanism, illustrating SPI data acquisition (Core 1) and TCP-based wireless transmission (Core 0) managed by FreeRTOS. (**d**) NeuroWireless platform schematic, featuring modules for data saving, communication, and visualization. (**e**) The user interface of the NeuroWireless platform, displaying real-time data visualization.

The overall architecture of the microsystem is illustrated in Figure 3a. This compact microsystem comprises three core components: a neural interface chip (RHD2132, Intan Technologies, Los Angeles, CA, USA), a main control FPGA chip (XC7Z020-2CLG400I, Xilinx, San Jose, CA, USA), and a 2.4 GHz Wi-Fi-capable wireless module (ESP32-WROOM-32E, Espressif, Shanghai, China). The RHD2132 amplifies and digitizes signals from 32 microelectrodes, converting them into 16-bit data and capturing raw neural electrophysiological signals. These signals are subsequently acquired by the FPGA via a Serial Peripheral Interface (SPI), where they are processed into spikes LFPs for real-time interpretation. The ESP32 module then reads the processed data from the FPGA over SPI and transmits the data wirelessly to a PC using the Transmission Control Protocol (TCP). Power for the entire microsystem is supplied by a lithium battery, with a power regulation module including four DC-DC converters (TLV62130, Texas Instruments, Dallas, TX, USA) that provide the necessary voltages for each component.

### 2.3. FPGA Signal Processing

The FPGA is programmed using Verilog HDL and SystemVerilog within the Vivado 2017.4 environment. Its primary function is to convert the raw data captured by the RHD2132 into neuron-specific spikes and population-level LFPs. This transformation not only aids in interpreting neural activity but also compresses the data, thereby reducing power consumption for wireless transmission. The signal processing workflow on the FPGA is illustrated in Figure 3b.

Within the SPI master core, the FPGA communicates with the RHD2132 via four Low-Voltage Differential Signaling pairs. Employing a round-robin fashion, it continuously cycles through commands to acquire 16-bit data from each of the 32 channels, sampled at 30 kHz per channel.

In the Finite Impulse Response (FIR) filter core, high-pass and low-pass FIR filters with 46 orders are implemented in parallel across all 32 channels. Designed with a Kaiser window and a cutoff frequency of 250 Hz, these filters optimize the computational load by exploiting coefficient symmetry, which reduces the number of adders and multipliers required in the digital signal processor (DSP) core for real-time digital filtering.

Following filtering, spike detection is performed on the high-pass signal path using a threshold-based approach. For each detected spike, 48 data points (corresponding to a 1.6 ms segment) around the threshold-crossing event are extracted to represent the spike waveform. On the low-pass path, the signal is down-sampled by a factor of 10 to yield the LFP.

Both spike and LFP data are temporarily stored in a First Input First Output. The FPGA frames each data packet with headers and channel numbers to organize the data for transmission. Using the SPI slave core, these framed packets are sent to the ESP32 module, enabling structured wireless data transfer to the backend system. By leveraging the FPGA’s high-speed parallel processing capabilities, this workflow efficiently extracts and processes neural signals in real time, ensuring data integrity and transmission efficiency.

### 2.4. Wireless Communication

#### 2.4.1. ESP32 Data Processing

The ESP32, developed in C++ within the Visual Studio Code 1.99 (VSCode) environment, is tasked with handling both SPI data acquisition from the FPGA and wireless data transmission to a PC via Wi-Fi using the TCP. Initial tests with a simple sequential approach—where data are read via SPI and then transmitted wirelessly—revealed a substantial reduction in transmission speed, resulting in critical packet loss.

To enhance transmission efficiency, we exploited the ESP32’s dual-core architecture and employed FreeRTOS for dynamic task scheduling, allowing parallel processing of SPI data acquisition and wireless transmission on separate cores without waiting for one task to complete before the other starts. Additionally, a dual-buffer approach is used to alternate data storage. Once the SPI task fills one buffer with data, the wireless transmission task immediately sends the fully populated buffer via Wi-Fi, while the SPI task continues writing data to the other buffer. Throughout this process, the statuses of the two buffers switch continuously as they alternate between data acquisition and transmission. The overall operating mechanism of the ESP32 is illustrated in Figure 3c. This parallel-processing approach optimizes the ESP32’s performance, fulfilling the stringent requirements for real-time, simultaneous wireless transmission of spikes and LFP data from all 32 channels.

#### 2.4.2. NeuroWireless Development

A PC software, NeuroWireless 1.0, was developed to interface with the wireless microsystem, enabling real-time data reception, signal visualization, and data storage. Based on Python 3.12, NeuroWireless provides a seamless workflow for wireless data processing, as outlined in Figure 3d.

The software first connects to the Wi-Fi network established by the ESP32 and configures the port and IP address settings. Once the connection is established, data packets are received over TCP. The Communication Module then parses the incoming packets to categorize signals as either spike or LFP. As shown in Figure 3e, within the Visualization Module, the parsed signals are passed from the backend to the UIThread, enabling the UI to update and display the signals in real time. Additionally, the Saving Module supports data storage in .NEV and .NS2 formats, facilitating further analysis using commercial software such as Offline Sorter. This integrated design ensures that NeuroWireless meets the demands of real-time signal processing and data management in experimental settings.

### 2.5. In Vivo Experimental Setup

A total of three male Sprague Dawley rats (250–300 g) were used in this study. Animals were housed in groups (one animal per Type IVC cage; 42 cm long, 28 cm wide, and 27 cm high) under a 12 h light 12 h dark cycle with food and tap water available ad libitum. All experiments were carried out with the permission of Beijing Association on Laboratory Animal Care and approved by the Institutional Animal Care and Use Committee at Aerospace Information Research Institute, Chinese Academy of Sciences (AIRCAS).

## 3. Results

### 3.1. Characteristics of PtNPs and PtNPs/PEDOT:PSS

**Figure 4:**
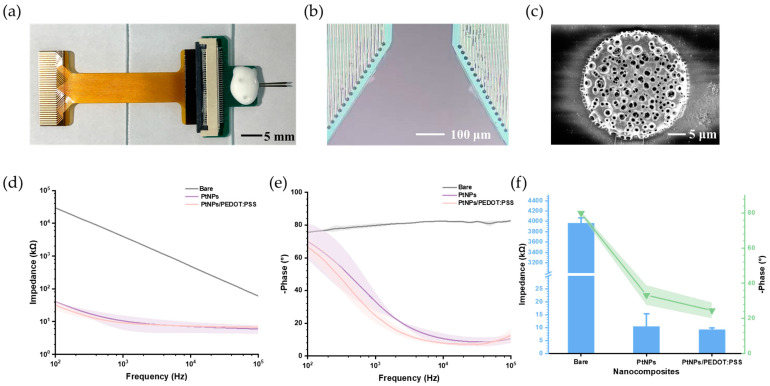
Images of the microelectrode array (MEA) and characteristics of modified microelectrodes. (**a**) Packaged MEA connected to the flexible printed circuit. (**b**) Microscopic image of the microelectrodes. (**c**) Scanning electron microscopy image of the microelectrode surface modified with PtNPs/PEDOT:PSS, showing the dense surface layer. (**d**) Impedance curves of bare, PtNPs-modified, and PtNPs/PEDOT:PSS-modified microelectrodes. (**e**) Phase curves of bare, PtNPs-modified, and PtNPs/PEDOT:PSS-modified microelectrodes (**f**) Comparison of impedance (blue) and phase (green) characteristics at 1 kHz among bare, PtNPs-modified, and PtNPs/PEDOT:PSS-modified microelectrodes. Data are shown as the mean ± SE.

Through design, fabrication, and modification, the MEA was optimized for neural electrophysiological signal detection. Figure 4a shows the prepared and modified MEA, which was encapsulated with a printed circuit board (PCB) via aluminum wire bonding and connected to the wireless microsystem through a flexible printed circuit. Figure 4b presents a microscopic image of the MEA modified with PtNPs/PEDOT:PSS, where all microelectrodes were coated with a black modification material. Scanning electron microscopy revealed a dense PtNPs/PEDOT:PSS layer covering the MEA surfaces (Figure 4c), significantly increasing the specific surface area.

To explore the conductivity characterization of PtNPs/PEDOT:PSS, electrochemical impedance spectroscopy was conducted in phosphate-buffered saline (pH 7.4) to compare the properties of bare, PtNPs-modified, and PtNPs/PEDOT:PSS-modified microelectrodes. As shown in Figure 4d,e, the impedance of the electrodes decreased substantially with increasing frequency, while the phase delay also decreased significantly. These results indicate that both PtNPs and PtNPs/PEDOT:PSS effectively improved the impedance and phase characteristics of the MEAs, with PtNPs/PEDOT:PSS showing superior performance.

Since the fundamental frequency of a spike is around 1 kHz, which is commonly studied in neuroscience research [38], the microelectrode properties at this frequency were further analyzed. At 1 kHz (*n* = 4) (Figure 4f), the impedance decreased from 3967.54 ± 100.06 kΩ (bare) to 10.49 ± 4.89 kΩ (PtNPs) and 9.16 ± 0.67 kΩ (PtNPs/PEDOT:PSS), while the phase shifted from −79.86 ± 1.14° (bare) to −33.22 ± 5.59° (PtNPs) and −24.52 ± 4.48° (PtNPs/PEDOT:PSS).

### 3.2. A Compact, High-Fidelity, and High-Throughput Wireless Microsystem

#### 3.2.1. Compactness

**Figure 5:**
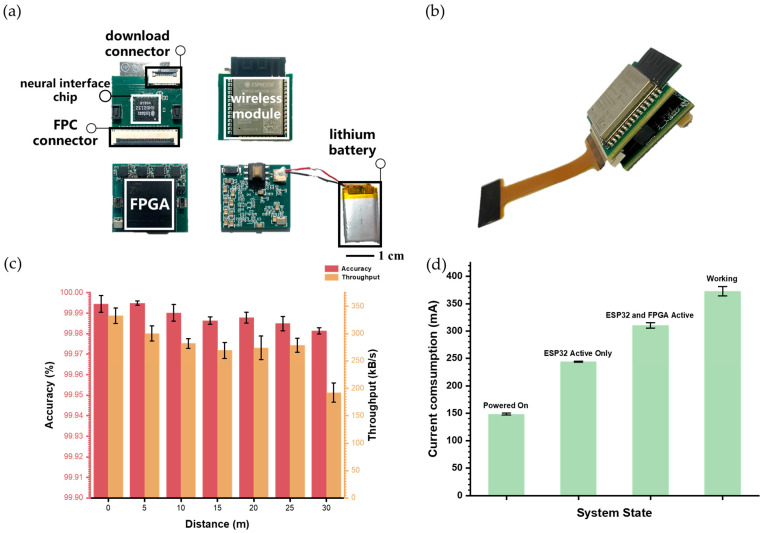
A compact, high-fidelity, and high-throughput wireless microsystem. (**a**) Front and back views of the wireless microsystem PCB alongside the lithium battery. (**b**) Fully assembled wireless microsystem connected to the flexible printed circuit. (**c**) Accuracy and throughput of wireless data transmission as a function of distance. (**d**) Current consumption of the wireless microsystem under different operational states. The data are shown as the mean ± SE.

The physical layout of the microsystem is shown in Figure 5a. The design employs a modular, dual-PCB configuration to optimize both functionality and compactness. The first PCB, a four-layer board, integrates the Intan chip and the ESP32 module, while the second, an eight-layer board, hosts the FPGA subsystem and the power regulation module. When assembled, the two PCBs are connected via two Board-to-Board (BTB) connectors, resulting in a compact form factor measuring 2.4 × 2.2 × 1.1 cm^3^ and a total weight of 8.68 g (Figure 5b). The microsystem is connected to the MEA on a separate PCB, with the MEA itself attached to the PCB through aluminum wire bonding.

#### 3.2.2. High Fidelity and High-Throughput

To ensure the feasibility and reliability of the wireless microsystem in practical applications, we evaluated its transmission range, accuracy, and throughput. Unlike tests that only assess the ESP32 wireless module, our evaluation measured the entire data path from FPGA to ESP32, and ultimately to a custom testing demo based on the NeuroWireless platform. This end-to-end testing allowed for a comprehensive assessment of the microsystem’s integrated performance.

Since the minimum transmission unit for both FPGA-to-ESP32 SPI communication and ESP32-to-PC communication is 8 bits, we generated random data packets containing values from 0 to 255 and stored them in the FPGA to represent a realistic range of possible transmissions. Accuracy was tested by evaluating the reception of a 1 MB dataset at intervals from 0 to 30 m (with 5 m steps) on the NeuroWireless platform. At 30 m, where software connection interruptions limited data reception, accuracy was calculated based on the successfully received data portion. Throughput was measured as the volume of data received over a 30 s interval at each distance.

As illustrated in Figure 5c, the microsystem maintained over 99.98% accuracy across the 0–30 m range, demonstrating stable performance with only a slight decrease in accuracy as distance increased—sufficient for the demands of real-world experimental conditions.

To support neural data recording, the microsystem must sustain a throughput of ~190.4 kB/s for LFP transmission (calculated as 3 kps at 65 bytes per sample). Assuming an average neuronal firing rate of 10 spikes per second, with spikes detectable on 32 channels, the required throughput for spike transmission would be approximately 30.6 kB/s (calculated as 10 spikes per second, 32 channels, and 98 bytes per spike). This gives an ideal overall throughput requirement of ~221 kB/s for optimal performance. Accordingly, the microsystem can stabilize operation within a 25 m range, meeting practical experimental needs.

These results demonstrate that our wireless microsystem can effectively support experimental data transmission within a 25 m range, offering high-fidelity transmission and robust throughput, thereby supporting a broad range of practical experimental applications.

#### 3.2.3. Current Consumption

To evaluate the power requirements of the wireless microsystem, we measured current consumption across four operational states—Powered On, ESP32 Active Only, ESP32 and FPGA Active, and Working—using a 3.7 V lithium battery as the power source. As shown in Figure 5d, current consumption increased progressively with the addition of each functional component, peaking at approximately 372 mA in the Working state during active data flow. At this peak consumption level, the microsystem can operate for approximately 40 min on a 250 mAh lithium battery, offering adequate runtime for most experimental scenarios while preserving a compact, battery-powered design that supports wireless functionality.

### 3.3. In Vivo Recording

**Figure 6:**
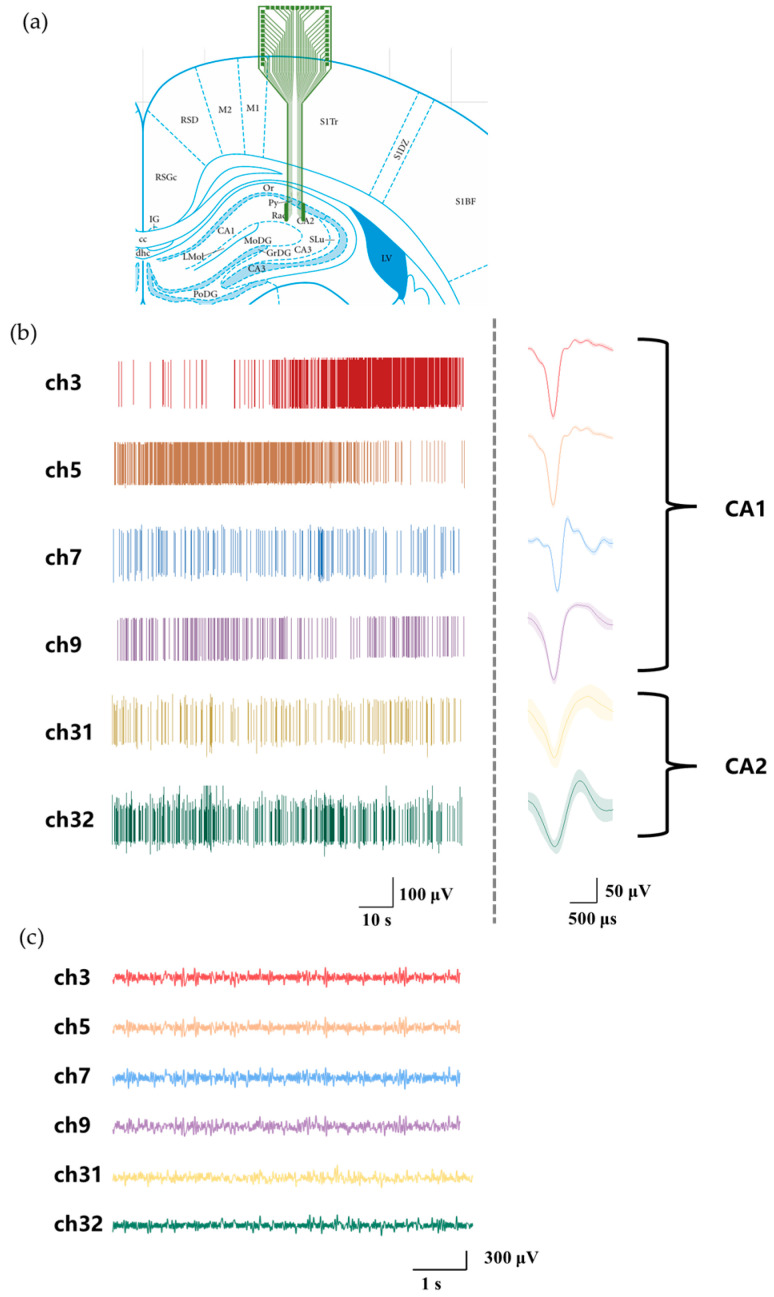
Neural signal recording from hippocampal CA1 and CA2 subregions. (**a**) Schematic illustration of the MEA implantation site. (**b**) Spikes and their averaged waveforms recorded from different channels. (**c**) LFPs recorded simultaneously across multiple channels.

As shown in Figure 6a, a dual-shank MEA was implanted into the CA1 and CA2 subregions of the rat hippocampus, and neural signals were recorded using the wireless microsystem. The left shank (ch1–16) captured signals from CA1, while the right shank (ch17–32) recorded signals from CA2. Figure 6b presents the detected multi-channel spikes alongside their corresponding averaged waveforms. Meanwhile, Figure 6c depicts the simultaneously recorded multi-channel LFPs. These results confirm the microsystem’s functionality, demonstrating its ability to perform real-time wireless acquisition of 32-channel neurophysiological signals. Additionally, the FPGA-based architecture facilitates parallel processing and separation of spikes and LFPs, underscoring the system’s advanced signal-handling capabilities.

### 3.4. Distinct Neural Activity Patterns Between Hippocampus CA1 and CA2

**Figure 7:**
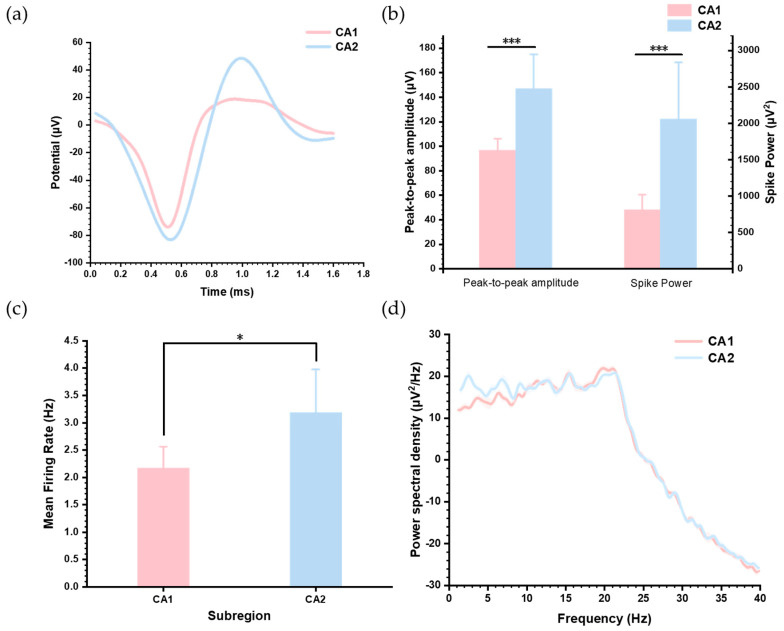
Comparison of neural activities between hippocampus CA1 and CA2. (**a**) Comparison of spike waveforms between the CA1 and CA2 regions. (**b**) Comparison of peak-to-peak amplitudes and power of spikes recorded from both subregions (*** *p* < 0.001). (**c**) Comparison of the mean firing rate of spikes in CA1 and CA2 (* *p* < 0.05). (**d**) Comparison of power spectral density of LFPs between the two subregions. The data are shown as the mean + SE.

To investigate differences in neuronal activity between the hippocampal subregions CA1 and CA2, we extracted and analyzed two typical neuron types from each subregion. Their spike waveforms are displayed in Figure 7a. To emphasize waveform differences, Figure 7b compares the peak-to-peak amplitudes and average power of the spikes, showing that neurons in CA2 exhibit higher discharge energy. Spike power was calculated as the mean of the squared amplitudes of all sampling points within each spike waveform. We further quantified the mean firing rates of these neurons, as shown in Figure 7c, which indicates that CA2 neurons fire at significantly higher frequencies than CA1 neurons. These results indicate heightened electrical activity in CA2 compared to CA1.

In addition, we analyzed the power spectral density (PSD) of LFPs recorded from the two subregions (Figure 7d). The results showed that in the 1–20 Hz frequency range, LFP power in CA2 was slightly higher than in CA1, while both subregions exhibited similar trends of power attenuation beyond 20 Hz. These observations highlight the enhanced neuronal and network-level activity in CA2 compared to CA1.

## 4. Discussion

In this study, we developed a wireless neurophysiological recording microsystem that integrates both hardware and software components. Its functionality was validated through in vivo experiments targeting the hippocampal CA1 and CA2 subregions, using silicon-based MEAs modified with PtNPs/PEDOT:PSS. The microsystem offers several notable features: it utilizes the parallel processing capabilities of an FPGA to enable real-time detection of spikes and LFPs across 32 channels sampled at 30 kHz; it achieves high-fidelity, high-throughput wireless data transmission over distances of up to 30 m; and it includes custom software, NeuroWireless, which seamlessly integrates with widely used commercial software. These capabilities make the microsystem well-suited for various animal studies, including those involving rodents and NHPs, offering researchers a practical and efficient tool for neurophysiological experiments. The wireless design, in particular, provides a significant advantage over traditional tethered systems by enhancing subject mobility, thereby expanding the range of behavioral and neurophysiological investigations that can be performed. Additionally, we observed that neural firing activity in the hippocampal CA2 subregion was significantly more pronounced than in CA1. This phenomenon, characterized by differences in spike amplitude, power, firing rate, and PSD of LFP, has not been previously reported.

Table 1 presents a summary of the performance parameters of our wireless microsystem in comparison with several notable works validated in vivo in recent years. Our wireless microsystem strikes a balance by offering a moderate number of recording channels, sampling rates, and resolution while maintaining a compact size and reasonable weight. Although the works in [22,29] support a greater number of channels, they do so at the cost of significantly reduced sampling rates. A key contribution of this work is the detailed evaluation of wireless data transmission accuracy and throughput across varying distances, providing specific and quantitative benchmarks that enrich the field’s understanding of such microsystems. Moreover, our microsystem integrates a custom-designed silicon-based MEA modified with PtNPs/PEDOT:PSS, paired with NeuroWireless, to enable seamless in vivo experiments.

However, several limitations and areas for improvement remain compared to previous works. One notable challenge is the relatively high power consumption of the wireless microsystem, which can lead to heat generation. This issue may cause discomfort to subjects and pose challenges for long-term animal experiments. Additionally, unlike prior studies such as [39], which incorporated features like multimodal signal detection, our microsystem currently lacks these functionalities.

In the future, we plan to optimize the design of the PCB, FPGA, and ESP32 components to lower the power consumption of the wireless microsystem, improve its integration, and boost data throughput. Furthermore, we aim to incorporate functionalities such as electrochemical sensing and stimulation to broaden the system’s capabilities. We also plan to explore advanced microelectrode materials such as dispersed carbon nanotube paper tape loaded with PEDOT for flexible supercapacitors with high energy density and deformation durability [40], and hydrogel-based microelectrodes for improved biocompatibility and mechanical conformity [41]. While initially designed for rodent models, the wireless microsystem has the potential to be adapted for use in NHPs and humans, enabling a wide range of neural signal acquisition tasks. This adaptability underscores its potential as a valuable tool in BCI research, neuroscience, and clinical applications across diverse fields.

## Figures and Tables

**Table 1:** Comparison of neural wireless microsystems.

Performance Parameters	Zhou (WAND) (2019) [22]	Keramatzadeh (2020) [29]	Bilodeau (2021) [17]	Shupe (NC3) (2021) [20]	Shin (2022) [16]	Lee (2023) [15]	This Work
Signal	Raw data	ECoG	LFP/spike	Spike	LFP/spike	ECoG	**LFP/spike**
Channels	96	8/36/72	32	16/32	16	9/15/24/32	**32**
Sampling rate (kHz)	1	1	20	5/10/20	8	30	**30**
Resolution (bits)	15	10	16	16	16	16	**16**
Size (mm^3^)	36 × 33 × 15	29 × 29 × 25	28 × 15 × 11	56 × 40 × 50	24 × 20 × 2	18 × 15 × 10	**24 × 22 × 11**
Weight (g)	7.4	27	1.7	40	2.44	2.1	**8.68**
Transmit distance	-	-	-	1 m	~50 m	-	**~30 m**
Accuracy	-	-	-	-	95% (50 m)	97%	**99.99%**
Throughput	250.88 kB/s	<256 kB/s	179.2 kB/s	-	<128 kB/s	1.92 MB/s (high mode)	**370 kB/s**
Consumption	172 mW	209 mW	37 mA	420/630 mW	74 mW	205.4 mA (high mode)	**350 mA**
Electrode	Commercially available	Gold	Commercially available	-	Pt black-modified	Gold	**PtNPs/PEDOT:PSS-enhanced**
Software	Python	-	-	MATLAB	-	Java	**Python, .NEV available**

ECoG, electrocorticography. LFP, local field potential.

## Data Availability

Data are contained within the article.
